# A qualitative analysis of the bud ontogeny of *Dracaena marginata* using high-resolution magnetic resonance imaging

**DOI:** 10.1038/s41598-018-27823-1

**Published:** 2018-06-29

**Authors:** Linnea Hesse, Jochen Leupold, Thomas Speck, Tom Masselter

**Affiliations:** 1 Plant Biomechanics Group and Botanic Garden, University of Freiburg, Freiburg, Germany; 2 Freiburg Center for Interactive Materials and Bioinspired Technologies (FIT), Freiburg, Germany; 3 Department of Radiology, Medical Physics, Medical Center University of Freiburg, Faculty of Medicine, University of Freiburg, Freiburg, Germany

## Abstract

The development of the branch-stem-attachment of *Dracaena marginata* was analyzed to clarify how a load-adapted arrangement of mechanically relevant tissues, i.e. the vascular bundles with fiber caps, is established during ontogeny. For this purpose, 3D images of four intact and developing buds of *D*. *marginata* were repetitively acquired *in vivo* within the time span of 180 days using high-resolution magnetic resonance imaging, as this method allows for non-invasive and non-destructive image acquisition. This methodical approach enabled the classification of distinct ontogenetic stages revealing the complex ontogeny of the branch-stem-attachment in *D*. *marginata* and the establishment of a load-adapted tissue arrangement within the junction between branch and main stem. This further allows for a first biomimetic abstraction and the transfer into a technical implementation of the form-structure-function principles found in branchings in *D*. *marginata*.

## Introduction

The branch-stem-attachments of arborescent monocotyledons and columnar cacti serve as a source of inspiration for the biomimetic optimization of technical fiber-reinforced ramifications^1–7^. In the first step of a biomimetic approach, the morphology, anatomy and biomechanics of junctions between the branch and stem have to be analyzed to understand their form-structure-function principles^8^. These can then be abstracted and transferred into technical fiber-reinforced branchings for automotive and aerospace constructions, sporting goods and architecture^2,9–12^. In the past, the gained knowledge on the mechanical properties of branchings was, however, often based on theoretical considerations (using simulations^6,11^) and two-dimensional analyses of the branching regions (by deformation analysis and anatomical evaluations of thin sections^1,3,13–15^). To overcome this limitation, a new method was developed on the basis of magnetic resonance imaging (MRI) enabling a non-invasive and non-destructive three-dimensional *in vivo* imaging of the branch-stem-attachments in *Dracaena marginata*^2,4,16^. While MRI is not a new imaging method in plant sciences^17–20^, its potential has been underestimated in the field of functional anatomy and biomechanics up to now. On the basis of MRI, it recently became possible to detect a load-adapted placement of mechanical relevant tissues within the branch-stem-attachment region in *D*. *marginata* by repetitively imaging a branch of an intact living plant under unloaded and subsequently under loaded conditions^2,16^. Hesse *et al*.^2^ could prove that the mechanical relevant tissues, i.e. the vascular bundles and their fiber caps, are mainly aligned in the direction of occurring stress trajectories. Depending on the orientation within the branch-stem-attachment these tissues withstand tensile loads on the adaxial region of the attachment^2^ or are abaxially pressed into the soft matrix of parenchyma tissues thus cushioning compressive loads^2^. But to date, one crucial question remains unanswered: How is this load-adapted tissue arrangement developed during the ontogeny of *D*. *marginata*?

A combined analysis of changing biomechanical and anatomical traits during ontogeny is enabled by the non-invasive and non-destructive character of MRI allowing for repetitive *in vivo* imaging of intact plants^18,20,21^. Different tissues are clearly definable since MR imaging also allows for a considerable tissue contrast^2^. As a consequence, the anatomy of the branch development in *D*. *marginata* can be studied non-invasively. In contrast, usual imaging techniques such as histological sectioning or micro-computer tomography (µCT) are destructive due to microscopic sectioning, sample trimming and ionizing radiation^2^. Thus, numerous samples of a plant structure at different ontogenetic stages would be required. Furthermore, plant specimens can only be tested either anatomically *or* biomechanically due to the strong invasiveness of these methods. And although Zimmermann and Tomlinson^22^ pointed out, that the development of cinematographic or motion picture analysis^23–27^ of a 3D structure reduces the required time of investigation from “*perhaps a year*” to “*a few days*”^22^, MRI of one sample takes only minutes to hours depending on the required resolution of the acquired images, by constant field strength, coil and signal to noise ratio (SNR).

In the present study, MRI was used to track the ontogenetic development of the *same* branches consistently over time, enabling the observation of the development of individual branch buds as a basis for defining distinct ontogenetic stages, which help understanding the ontogeny of branches in *D*. *marginata*. Furthermore, the development of the load-adapted arrangement of mechanical relevant tissues could be studied and discussed, providing the basis for a biomimetic optimization of fabrication processes of technical fiber-reinforced branchings and consequently leading to the optimization of technical fiber-reinforced branched structures.

## Results

### General morphology and development of the specimens

**Figure 1:**
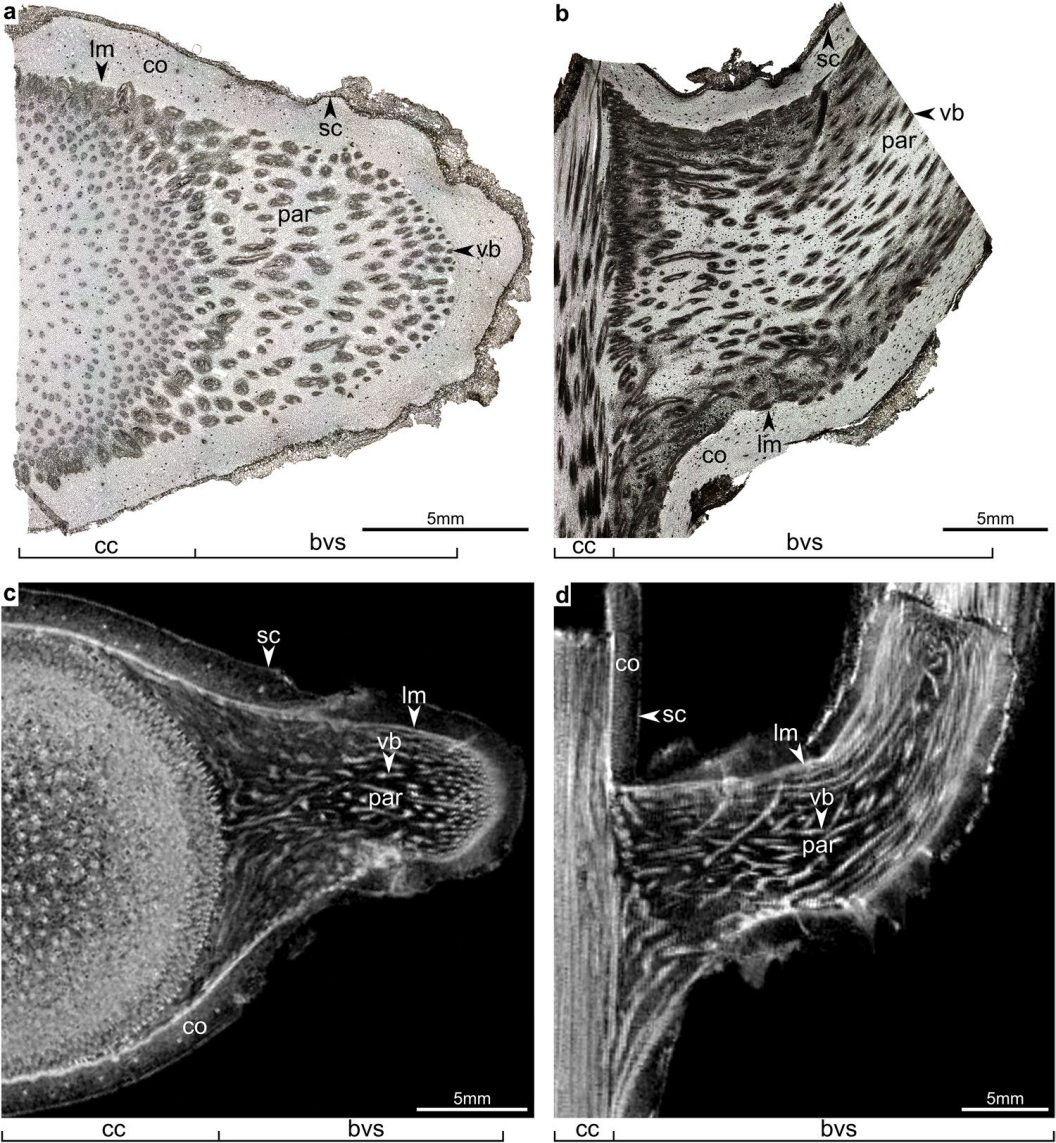
Light microscopy (**a** and **b**) and magnetic resonance (**c** and **d**) images of the branch-stem-attachment of *Dracaena marginata* for comparison of the imaging methods. bvs: vascular system of the branch; cc: vascular bundles of the central cylinder of the main stem; co: cortex; lm: lateral meristem; par: parenchyma tissue; sc: storied cork; vb: vascular bundles with fiber cap.

Although MRI of single cells or cell clusters is feasible with dedicated micro-coils, the sensitivity of MR does not allow resolution on cellular level of bulk plant material as larger samples requiree for large RF-coils. However, the resolution of MRI allows for the analysis of ontogenetic changes in tissues. This can be proven by a comparison of light microscopy images with MR-images of a transversal (axial) and longitudinal (sagittal) section of the branch-stem-attachment of *Dracaena marginata* (Fig. 1). In MRI, various tissues easily can be detected due to a pronounced tissue contrast. These include the vascular system (entity of vascular bundles and fiber caps) and all meristematic tissues (a detailed explanation of the terminology of monocot meristems is given in the ‘Material and Methods’ section), the primary thickening meristem (PTM), as well as the secondary thickening meristem (STM) and the storied cork, which are clearly visible due to their bright appearance (high pixel intensity) in T_2_^*^- weighted^2^ MR images (Fig. 1c,d). Cortex and inner parenchyma tissues appear darker (due to low pixel intensity), while the cork tissue towards the stem periphery almost fully disappears (Fig. 1c,d). This is in contrast to light microscopy images, where the meristematic tissues are much more difficult to detect (Fig. 1a,b). In the following, the authors combined PTM and STM in the lateral meristem ‘lm’ (Fig. 1c,d), as the transition between PTM and STM proved to be continuous.

After separating (further referred to as decapitation) the apical region of the main stem bearing older branches from more basal regions, four buds located below the decapitation site emerged. The individual development of each examined bud (B1–B4) is comparable. However, the chronological occurrence of distinct developmental features (Table 1) can differ in detail and depends on the position of a bud along the stem axis. The developmental features in Table 1 are explained in detail in the following sections.

**Table 1:** Summary of distinct developmental features (feat.) for every delimited ontogenetic stage (OS1–7) and each considered bud (B1–B4).

OS	OS1						OS2		OS3		OS4		OS5						OS6	OS7
day	0						0–15		15–20		20		25–90						90–180	180
feat.	lt1	lt2	bt1	bt2	bt3	bt4	m exp	m con	m spl	bump	dif ip	vis VB	mc	am	con apex	VB lay.	crack prop	lp	vertical	elong
B1	+	+	+	+	+	−	+	+	+	+	/	/	/	/	/	/	/	/	/	/
B2	+	+	+	+	+	−	+	+	?	+	+	+	+	+	+	+	+	+	+	+
B3	+	+	−	+	+	+	+	+	?	+	+	+	+	+	+	+	+	+	+	−
B4	+	+	+	+	+	−	+	+	−	−	−	−	−	−	−	−	−	−	−	−

The duration of each ontogenetic stage (day of exp.) is given for bud B2, which showed the most complete and fastest development of the four studied buds. B1 deceased (/) before entering OS4. B2 becomes dominant and partly (B3) or fully (B4) re-imposed inhibition to the following buds. The meristem splitting (m spl) was not depicted for B2–B3 and is assumed (?) to have taken place between day 10 and day 15 of the experiment. +: If a developmental feature was present; −: if a developmental feature is absent; /: if a bud deceased; (?): if a developmental feature is unclear because it has not been depicted; am: delimitation of the apical meristem becomes possible; bt1–4: bud traces; bump: developing bud appears as surface bump; con apex: the concave apex is developed; crack prop: crack propagation of outer tissues; day: day of image acquisition); dif ip: differentiation of the inner meristematic pole; elong: onset/continuation of elongation/maintenance growth of the branch; feat.: specific developmental feature; lp: leaf primordia appear at stem surface; lt1–2: leaf traces; mc: visibility of meristematic cap; m con: connection of bud meristem and secondary thickening meristem (STM) of the main stem; m exp: meristem expansion; m spl: splitting of bud meristem and STM; VB lay.: layered organization of the vasculature in the branch-stem-attachment becomes clear; vis VB: visibility of developing vascular bundles; vertical: branch changes from a preliminary horizontal orientation to a vertical orientation.

**Figure 2:**
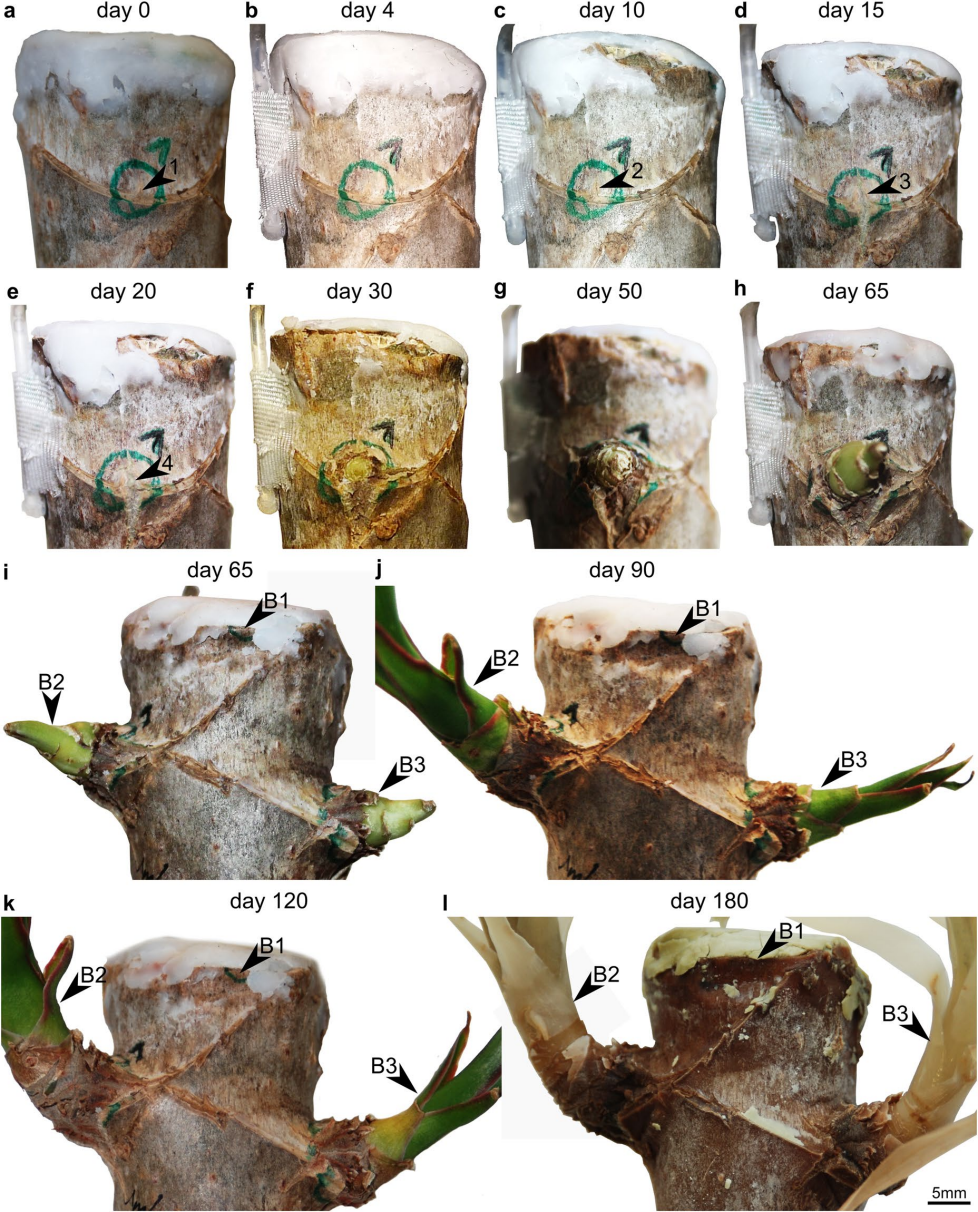
Development of buds B1–B3 of *Dracaena marginata* from the moment of decapitation at day 0 (**a**) until the end of the experiment at day 180 (**h**). Bud B4 is located at the back, i.e. outside of the imaged stem surface, and thus is not visible. The secondary protective tissues (periderm) of the main stem become ruptured during the development of bud B2 (**a**–**h**). Once the buds reach the stem surface, the leaves enlarge and the branch changes its initially horizontal orientation to a vertical one. Bud B1 dies off at day 20 due to lethal drying effects at the neighboring decapitation site. Bud B2 becomes dominant after this event and shows a higher degree of development then bud B3. Arrows 1–4 highlight the crack propagation indicating that bud B2 penetrates the secondary protective tissues.

Of these most apical four buds, only the buds B2 and B3 fully developed (Fig. 2i–l). Furthermore, the imaged buds B1–B4 competed for dominance or re-imposed inhibition. This became increasingly pronounced with ongoing ontogeny of the buds. The most apical bud (B1; Fig. 2i–l) died off at day 20 due to lethal drying effects at the neighboring decapitation site. Thus, the subsequent bud (B2) became dominant and partly re-imposed inhibition to the neighboring bud B3 (Fig. 2i–l) and fully inhibited growth of bud B4 at day 25.

In dormant state, both buds and leaf traces could be easily detected on the outer surface of the stem (arrow 1 in Fig. 2a). The germination of a renewal bud first became visible through the formation of a swelling at day 10 (Fig. 2c). Crack propagation or splitting of the cortex and cork occurred with progressive bud growth (arrows 2–3 in Fig. 2c,d). Subsequently, the periderm split fully in longitudinal direction of the main stem and leaf primordia appeared at its surface (arrow 4 in Fig. 2e).

### Bud anatomy at day of decapitation (day 0)

**Figure 3:**
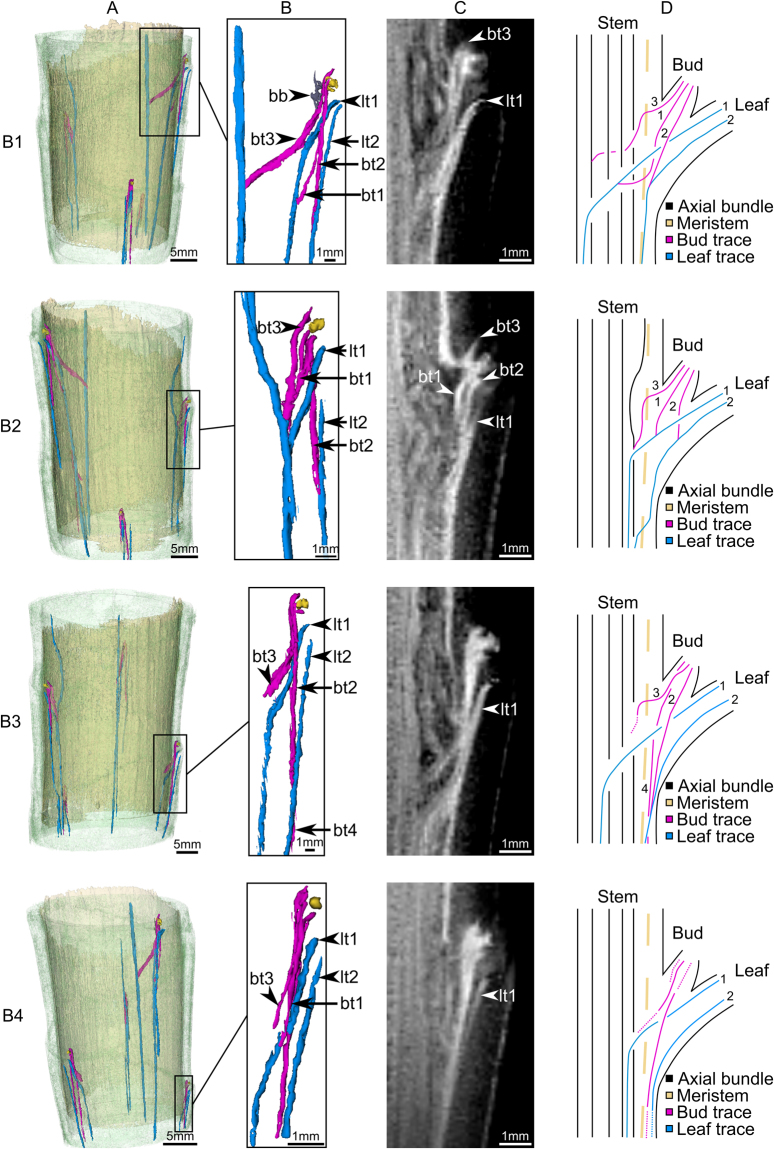
Bud anatomy of *Dracaena marginata* at day of decapitation (day 0) acquired via MRI. Rows: images and schematic drawings of buds B1–B4, respectively. Columns: (**A**) 3D data representation (3D models) of the stem segment with the stem surface (green), the lateral meristem and bud meristem (yellow), the axial bundles and leaf traces (blue) and the bud traces (pink). (**B**) Detailed overview of the 3D models of leaf traces (blue; lt1–2), the bud traces (pink; bt1–4) and bud meristem (yellow) of each bud. Bridge bundles (bb) have been highlighted in grey for bud B1. (**C**) Sagittal image of the underlying magnetic resonance data of each respective bud. (**D**) Schematic drawing in the style of Zimmermann and Tomlinson^22,32^ for a better understanding of the bud anatomy. Bud traces (bt1–4) and leaf traces (lt1–2) are labeled by numbers (1–4) and (1–2), respectively. Dotted lines were used for parts of a course or connection of a bundle (bt or lt) that could not be determined unequivocally.

Although the anatomy of each bud differs in detail, general similarities can be found and described. Each bud is accompanied by two leaf traces (lt1 and lt2 in Fig. 3, Table 1), which supply the bud with two bud traces (bt1 and bt2 respectively; Fig. 3, Table 1). The leaf trace lt1 always approaches a bud after an acute basal deviation from the central cylinder towards the periphery. It leads to the stem surface under and close to the bud (adaxial side of the leaf) and is visible from the outside in the leaf scar directly under the minute axillary bud. The leaf trace lt2, in contrast, crosses the lateral meristem of the main stem at a larger distance below the bud. It then runs in longitudinal direction through the cortex, releasing an accompanying bud trace (bt2, Fig. 3) and distally is running in parallel to lt1. The bud trace (bt1) is always a derivate of leaf trace lt1 and runs directly into the bud center. The bud trace (bt2) is always a derivate of the leaf trace lt2 and runs straight upwards towards the left or right side of the bud. The origin of a third bud trace (bt3; Fig. 3, Table 1) can be assigned to major, intermediate or minor axial bundles and thus is linked to the inner vascular system of the central cylinder (major bundle in bud B1 in Fig. 3). A fourth bud trace (bt4) can only be detected in bud B3 (bt4 in Fig. 3, Table 1) while bt1 of this bud cannot be identified. The connectivity of bt4 in basal direction to the main stem cannot be observed, as it is located outside of the field of view.

### General bud ontogeny determination of distinct ontogenetic stages

The examined developmental features (Table 1) of each bud (B1–B4) allows for classifying distinct ontogenetic stages (OS1–7) that may help clarifying the general bud ontogeny of *Dracaena marginata*. The occurrence of a specific ontogenetic stage varies with the position of the bud along the stem. Thus, the following headers specify the duration of each ontogenetic stage for bud B2 (also see Table 1), which showed the most complete and fastest development of the four studied buds.

#### Ontogenetic stage OS1 (day 0)

**Figure 4:**
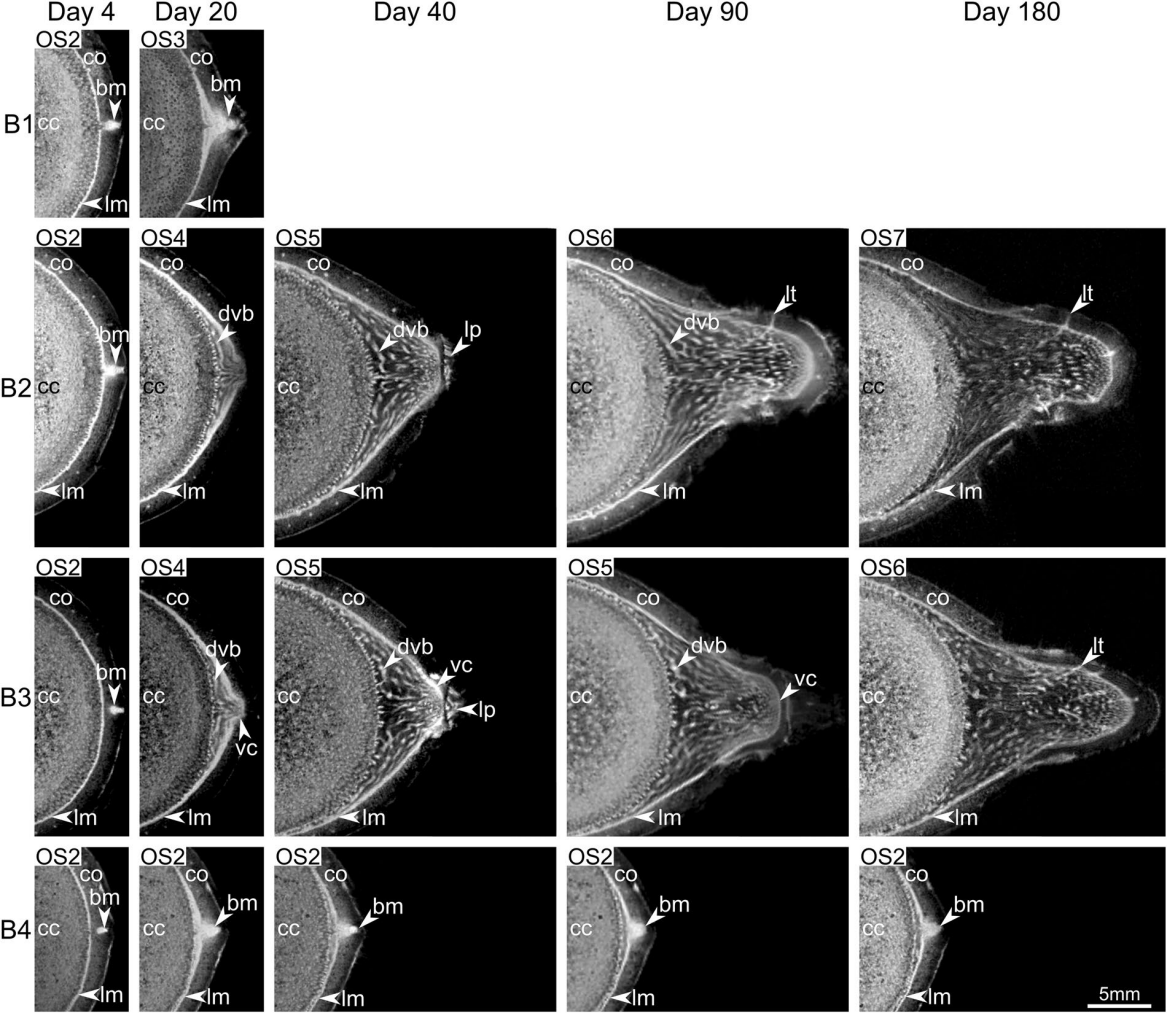
Axial MR images of the bud ontogeny in *Dracaena marginata*. The axial images are located in close proximity to the dotted line in Fig. 6. Rows: images of buds B1–B4 respectively. Columns: day 4-day 180 of image acquisition after decapitation. Bud B1 died back after 25 days and is therefore missing in the columns of day 40-day 180. The ontogenetic stage (OS2–OS7) for each individual image is given. bm: bud meristem; cc: central cylinder; co: cortex; dvb: developing vascular bundle; lm: lateral meristem; lp: leaf primordium; lt: leaf trace; vc: apical vegetative cone of the branch.

**Figure 5:**
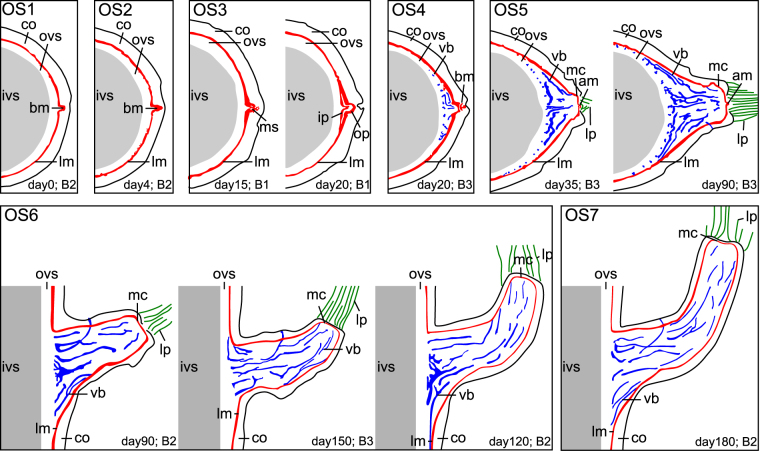
2D segmentation images as schematic representation of the identified ontogenetic stages OS1–OS7. am: apical meristem; bm: bud meristem; B1–B4: observed buds; co: cortex; ip: inner pole, ivs: inner vascular system; lm: lateral meristem; lp: leaf primordia; mc: meristematic cap; ms: meristem splitting; op: outer pole; ovs: outer vascular system; vb: vascular bundle.

OS1 characterizes the situation found in buds directly after decapitation (day 0; Fig. 3, Table 1). In transversal plane the bud either appears as a V-shaped protrusion of the lateral meristem (basal bud region; B2 at day 4 in Fig. 4, OS1 in Figs 5 and S2a) or is represented as peripheral tissue complex within the cortex showing high pixel intensities (apical region of the bud; B3-B4 at day 4 in Figs 4 and S4a–S6a). Thus, the bud apex is located within the cortex and is initially not in contact with the lateral meristem. The anatomy of a bud directly after decapitation (day 0 or OS1, Fig. 3) is described in detail above.

#### Ontogenetic stage OS2 (days 0–15)

**Figure 6:**
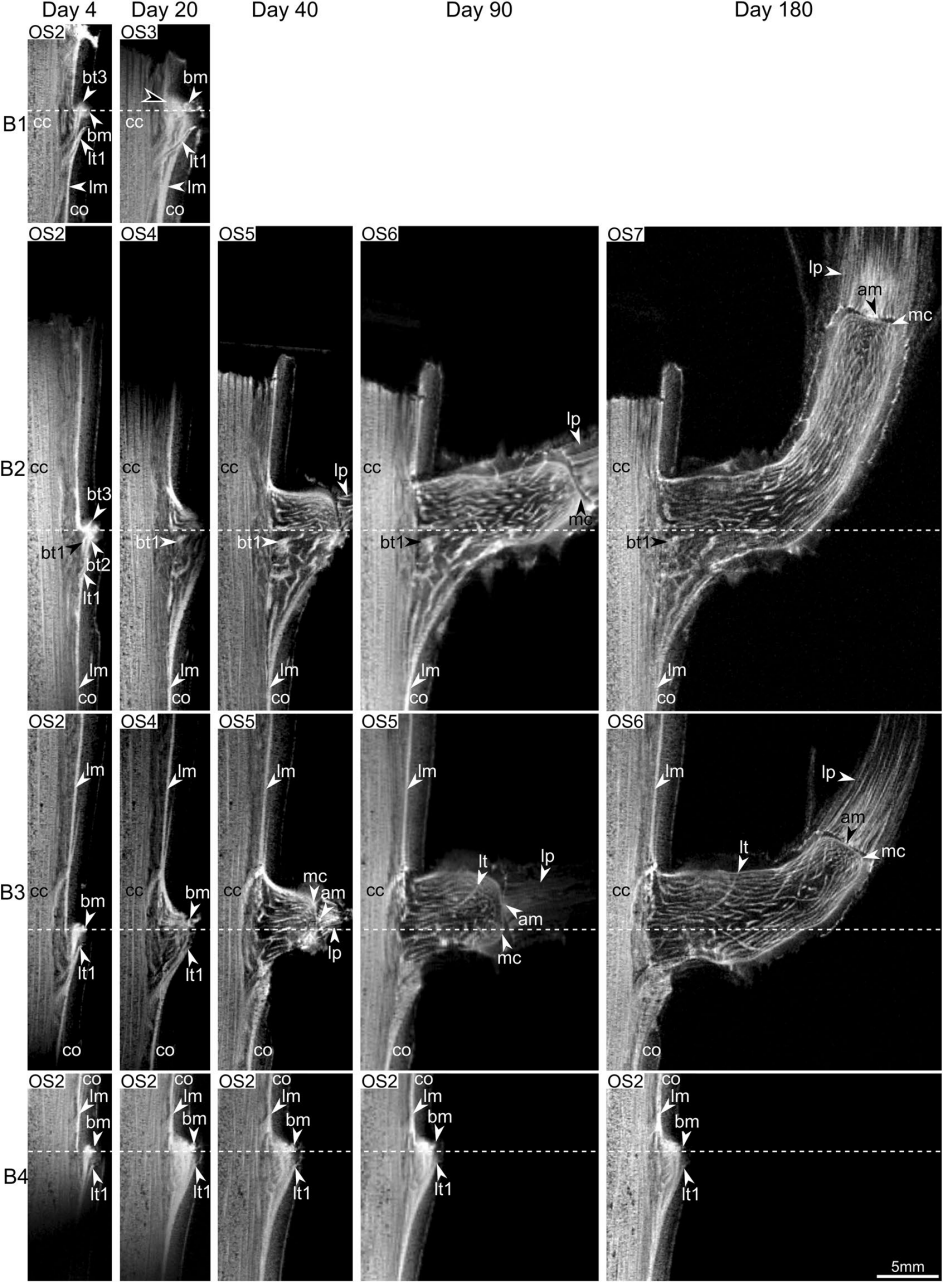
Sagittal MR images of the bud ontogeny in *Dracaena marginata*. Rows: images of buds B1–B4 respectively. Columns: day 4-day 180 of image acquisition after decapitation. Bud B1 died back after 25 days and is therefore missing in the columns day 40-day 180. A reference line is placed into the center of the bud meristem (bm), splitting the vascular bundles of the branch into adaxial bundles which run in the branch perpendicular to the main stem and abaxial bundles describing a curvature arrangement from the main stem and into the branch towards the branch apex. The ontogenetic stage (OS2–OS7) for each individual image is given. The white framed arrow in the image of B1 at day 20 highlights the fusion of bud meristem (bm) and lateral meristem (lm). am: apical meristem; bm: bud meristem; bt1–3: specific bud traces (also see Fig. 3); cc: central cylinder; co: cortex; lm: lateral meristem; lp: leaf primordium; lt: leaf trace; lt1; specific leaf trace; mc: meristematic cap.

Apical dominance is released within the first four days after decapitation and the development of renewable buds is activated directly below the section plane. This is the onset of OS2 and is characterized by an expansion of the bud meristem in buds B1–B4 (day 4 in Figs 4–6 and S1–S7, Table 1). The bud meristem expands within the cortex of the main stem as its cells re-embryonalize (dedifferentiate). The cell proliferation is visualized as dedifferentiating cortex cells change their pixel intensity from darker grey values to brighter pixels matching the pixel intensity of typical meristematic tissues (PTM, STM, apical meristem and storied cork). Already existing tissues of the bud such as leaf and bud traces are maintained, yet concealed by the resulting ‘partial volume effects’ (see Material and Methods), which lead to a blurry appearance of the budding region (day 4 in Figs 4 and 6). OS2 begins shortly after decapitation (day 0) and can last up to 15 days after decapitation, dependent of the developmental speed of the bud (B1 in Fig. 5 and B1 and B4 in Supplementary Figs S1 and S6 and S7). The prolonged extension of the meristem eventually results in a contact of the bud meristem (PTM) with the lateral meristem of the main stem (STM, Table 1).

#### Ontogenetic stage OS3 (days 15–20)

Once the bud meristem has established contact to the lateral meristem of the main stem (OS2), both meristems split again (B1 at day 15–20 in Fig. 4 and OS3 in Figs 5 and S1, Table 1) resulting in an inner pole, adjacent to the vascular system of the central cylinder and an outer cortical pole, marking the onset of OS3 (Fig. 5). The splitting occurs within the cluster of dedifferentiated cells of the expanding meristem and is not restricted to a single plane. By this, a three-dimensional attachment of the developing branch to the main stem is established. As the outer pole remains meristematic in later ontogenetic phases it further produces branch tissues and links the branch to the main stem. The tissue between the two meristem poles gradually differentiates and thus appears darker in MR images (B1 at day 20 in Fig. 4). Tissues that were concealed by the meristem expansion reappear (B1 and B4 at day 20 in Fig. 6). First procambial strands are determined between the splitting inner and outer poles in this phase of bud/branch development.

#### Ontogenetic stage OS4 (day 20)

The interior part of the meristem, the inner pole, fully differentiates in OS4 and single vascular bundles become visible (OS4 in Fig. 5, Table 1). In contrast, the peripheral outer pole stays meristematic and undifferentiated (B2 and B3 at day 20 in Fig. 4, OS4 in Figs 5 and S2d,e and S4d,e).

#### Ontogenetic stage OS5 (days 25–90)

**Figure 7:**
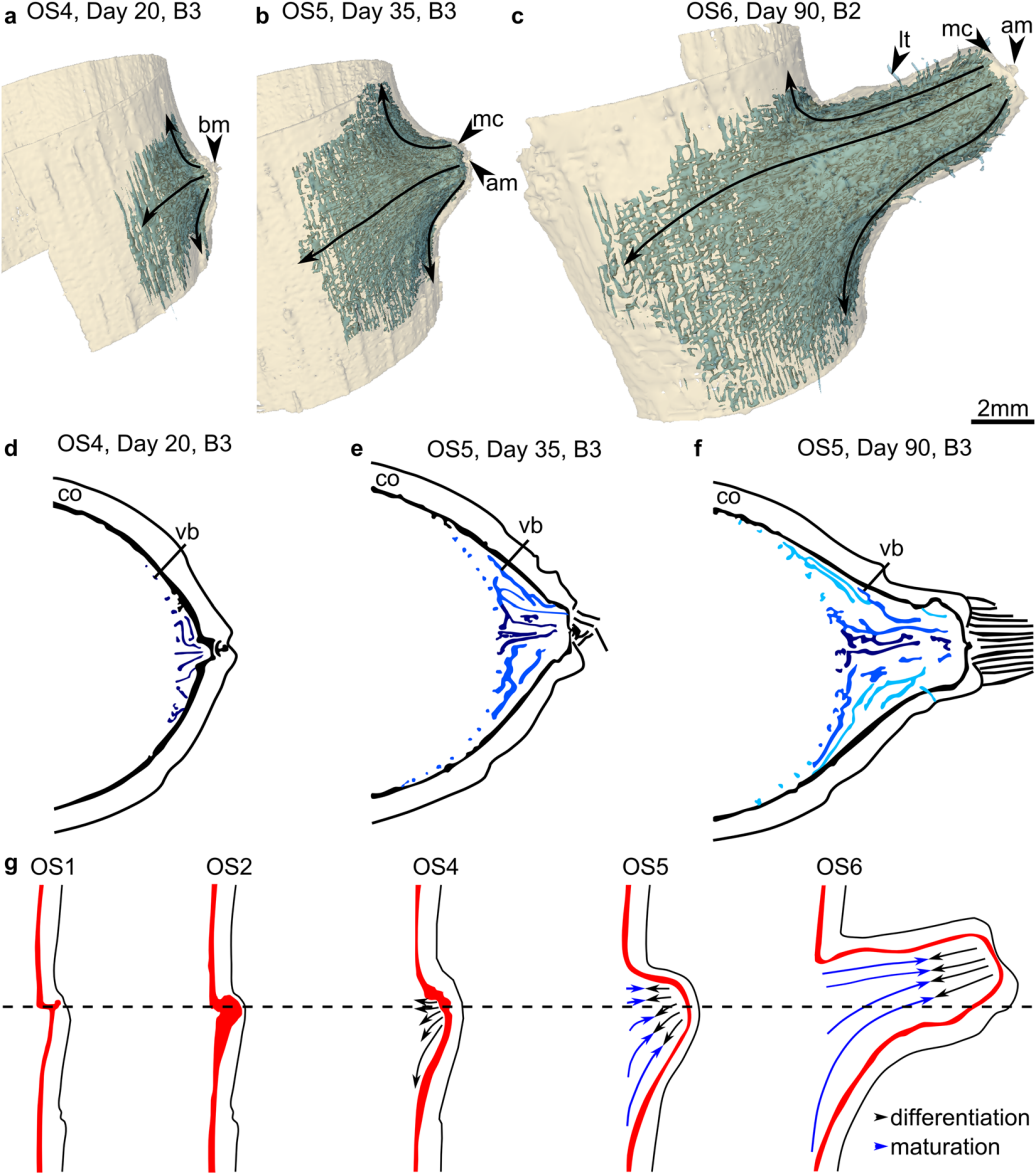
Arrangement of vascular bundles in the branch-stem attachment of *Dracaena marginata*. (**a**–**c**) 3D data representations of the development of meristematic tissues (yellow) and vascular bundles with fiber caps (blue) involved in the branch ontogeny at OS4 to OS6. The apical vegetative cone with meristematic cap (mc) and apical meristem (am) are gradually developed. The black arrows indicate the direction of the 3D attachment of vascular bundles of the branch to the main stem. (**d**–**f**) Schematic drawing of the establishment of the multi layered arrangement of vascular bundles highlighted by an intensity gradient of the color blue with innermost vascular bundles colored in dark blue and outermost vascular bundles colored in bright blue. (**g**) Schematic drawing of the development of the vascular system of a branch. The black arrows within the branch indicate the direction of tissue differentiation while the blue arrows indicate the direction of cell maturation. The dotted line splits the vasculature of the bud in adaxial bundles which attach perpendicular to the main stem and abaxial bundles which describe a curvature running up the main stem into the branch. bm: bud meristem, co: cortex; lt: leaf trace, vb: vascular bundles with fiber caps.

The vegetative crown of the branch fully develops in OS5, which is characterized by five interrelated steps: (1) meristematic cap and apical meristem become visible (B3 at day 35 in OS5 of Figs 5 and 7b; for details also see day 25 in Supplementary Fig. S5f, Table 1). (2) The vegetative crown of the branch progressively enlarges during this process to a flattened or concave apex as typical for monocots (B2 and B3 at day 20–90 in Figs 6 and 7 and day 20–65 in Supplementary Figs S4e–l and S5e–l, Table 1). This is the result of a rapid primary thickening growth (establishment growth) via the PTM of the branch in respect to its elongation^28–31^. The final width of the meristematic cap is accomplished between days 65 and 90 (for details see Supplementary Figs S3j–m and S5j–m, Table 1). (3) Vascular bundles develop at the outer margins of the branch attachment region, run around the main stem and finally into the branch causing a layered arrangement of vascular bundles within the branch-attachment-region (B2-B3 at days 20–180 in Fig. 4, OS 5 in Figs 5 and 7a–c, Table 1). Figure 6 additionally shows, that the bud meristem (bm in Fig. 6 at day 4) divides the developing branch in an upper adaxial side of the branch showing an increasingly perpendicular (to the main stem) attachment of branch vascular bundles, and a lower abaxial side with branch bundles describing a curvature running from the main stem towards the branch apex (dashed line in Fig. 6). (4) Outer tissues of the main stem rupture, allowing the branch to appear at the stem surface (Fig. 2c–h). (5) Young leaf primordia at the branch apex become clearly visible from the outside of the stem and mature continuously with time (Fig. 2h–j, Table 1).

#### Ontogenetic stage OS6 (days 90–180)

The branch changes its preliminary horizontal orientation to a vertical orientation (B2-B3 at days 90–180 in Figs 5 and 6; for details also see Supplementary Figs S3m–p and S5m–p, Table 1). This shift of the growth direction takes place mainly in the first centimeter of the branch apex (OS6 in Fig. 5; for details also see Supplementary Figs S3m–p and S5m–p). Consequently, the angle between branch and main stem changes in this ontogenetic stage from 92.3° +/− 9.3° (n = 8) to 29.0° +/− 15.8° (n = 5).

#### Ontogenetic stage OS7 (starting from day 180)

Once the ontogenetic stage OS7 has been reached, the branch progressively elongates (Table 1).

## Discussion

The initiation of the examined axillary buds in *Dracaena marginata* takes place at a very early stage of the development of the main stem in close proximity of its apical meristem. Tomlinson and Zimmermann described this early bud initiation in monocots and identified different types of bud attachments dependent of the time of bud initiation and with this of its distance from the apical meristem of the main stem^22,32^. In the present study, the ontogenetic stage OS1 describes the anatomy of a bud at the day of decapitation (day 0). A change in bud anatomy does not occur until the next developmental stage (OS2) four days after decapitation. The bud anatomy at OS1 therefore reflects the type of primary bud structure and attachment at bud initiation and allows comparison to the bud attachment types defined by Zimmermann and Tomlinson^22,32^.

Of the three identified bud traces (bt1–3 Fig. 3, Table 1), the traces bt1 and bt2 originate from two respective leaf traces (lt1–2 in Fig. 3, Table 1) and are therefore satellite bundles^22,24,31,32^. Zimmermann and Tomlinson termed the resulting type of initial bud attachment an ‘indirect or satellite attachment’ (Type C of vascular branching^22,32^). The origin of a third bud trace (bt3; Fig. 3) can in contrast be assigned to major, intermediate or minor axial bundles and thus is restricted to the inner vascular system of the central cylinder (major bundle in bud B1 in Fig. 3) reflecting the initial developmental stage in which dormant buds remain. Whether the third bud trace (bt3) directly inserts the bud as an axial bundle (Type B of vascular branching^22,32^) or as a diversion of an axial bundle (Type D of vascular branching^22,32^) could not be answered satisfactorily by the results of the present study. Yet, Zimmermann and Tomlinson have observed both axial bundles and satellite bundles in the axillary buds of *Dracaena fragrans* (B and C type of bud attachment^22^ p. 148^31^, p. 487]). Hence, it can be hypothesized that the bud trace bt3 could be an axial bundle in *D*. *marginata* as well. Further bud traces can occur (bt4 of bud B3 in Fig. 3, Table 1).

The bud development following the initial attachment is at first inhibited due to apical dominance. This inhibition is released by decapitating the examined specimen several months after the examined buds had been initiated. The further development of all four buds is comparable despite differences concerning the individual speed of development and initial primary attachment, as they are located consecutively within a restricted area along the main stem. This is in accordance with Tomlinson and Zimmermann^33^ who have stated that the attachment of lateral organs can be similar if their “development is the same”. In the present study the bud development is analyzed several months after bud initiation. At that later time of analysis, a secondary vascular system (outer vascular system^22^) has already developed in the main stem. In addition to the early bud attachment to the primary vascular system of the stem (initial primary attachment), the developing bud is attached to the outer vascular system of the main stem. In ontogenetic stage OS2 this late attachment is initiated by the establishment of a contact of the bud meristem (PTM) with the STM of the main stem through the expansion of the bud meristem within the cortex. The following meristem splitting (OS3) is the key to the late bud attachment as a connection between primary provascular strands of the branch and the secondary vascular bundles of the main stem is established. Zimmermann and Tomlinson have mentioned physiological poles or growth centers to describe the differentiation of vascular strands between leaf primordia or branches and the vascular system of the shoot within the monocot crown^22,27,32,34^. In the present study, the portions of the split meristem in OS3 – the bud meristem (PTM; outermost pole) and the STM (innermost pole) of the main stem – are forming the growth centers. The direction of vascular differentiation progresses from the outermost pole (bud meristem or PTM) to the inner most pole (STM of the main stem), which is indicated by the dedifferentiation processes visualized in OS2 (days 4–20 in Fig. 4, OS2–OS3 in Figs 5 and 7g). In contrast, the maturation of procambial strands to vascular bundles with fiber caps progresses from the inner pole (STM) to the outer pole (bud meristem or PTM; Fig. 7g). Thus, vascular strands are differentiated between a primary meristem of the bud (PTM and apical meristem) and a secondary thickening meristem of the main stem (STM).

In developmental stage OS4 the connectivity of the vascular system of branch and main stem is established by tissues originating from the innermost pole. This pole is “used up” during this process while the outermost growth center, the bud meristem, remains meristematic (Table 1). The bright inner meristem band (B1 at day 20 in Fig. 4 and OS3–OS4 in Fig. 5) disappears within the MR images (B2–B3 at day 20 in Fig. 4). It appears that a complete fusion of bud meristem and lateral meristem did not occur in OS2 and both meristems remained separate at all times. Their connection, however, is the crucial developmental step for attaching the procambial strands of the bud to differentiated but not fully maturated vascular bundles of the outer vascular system of the main stem in OS4 (see Fig. 2 in Haushahn *et al*.^15^ for a light microscopy image of a mature bundle attachment). It explains why the branch is only connected to outer stem tissues of the main stem resulting in a “flange-mounted” type of attachment of the branch^3,10,15,16^. The attachment area of the branch to the main stem increases due to mechanical (increased mass of the branch) and physiological (increased water need and uptake) constraints. This is achieved by the lateral meristem in the margins of the branch-attachment region (days 20–180 in Figs 4 and 6, Supplementary Figs S1–S7) where the newly developed vascular bundles are connected to the outer vascular system of the main stem ensuring vascular connectivity to the main stem. As a result, the vascular bundles are connected to the secondary vascular system in the periphery of the main stem. As a consequence the vascular bundles supplying and mechanically anchoring the branch run around the main stem following a path towards the center and the apex of the branch (Fig. 7d–f). This leads to a multi-layered arrangement of the vascular bundles. Those developed at an earlier time of bud development are located within an inner region of the branch-stem-attachment (Fig. 7a,d) whereas bundles developed later (Fig. 7b,c,e,f) are placed towards the periphery of the branch displaying a flange-mounted appearance within the branch-attachment-region (Figs 5 and 7 ^3,10,15,16^.

Furthermore, new vascular bundles are being developed at the inner surface of the lateral meristem (see dvb in Fig. 4) and are increasingly oriented perpendicular to the stem at the adaxial side of the branch (B2–B3 in Figs 6 and 7g). They are thereby placed in a load-adapted manner as they are being aligned in direction of tensile strains caused by the increasing weight of the branch. This results in a high tensile resistance within this region^2^. In contrast, within the abaxial side of the branch vascular bundles describe a curvature. This also can be interpreted as a load adaptation which allows for “cushioning” compressive strains as vascular bundles are being pushed into the visco-elastic parenchyma tissue during mechanical loading of the branch^2^. This placement of vascular bundles additionally seems to prevent lethal failures caused by buckling of the vascular bundles with fiber caps due to compressive stress^2,35,36^. This failure could be lethal as the vascular bundles of monocots lack vascular cambia which prevents a local restoration of damaged vascular tissues. In summary, the individual branch ontogeny directly at the branch-stem-attachment region seems to follow the laws of stress distributions in typical cantilever beams under transverse loading.

The observed transition of the horizontal branch orientation towards a (nearly) vertical orientation is in accordance to previous assumptions made on the ability of leaning or horizontal monocot stems restoring their vertical position^37–40^. The change in orientation of the branch is accomplished through a (slight) asymmetric increase of primary growth on the abaxial side of the branch (compare days 90–180 of B2–B3 in Fig. 6 and OS6 in Fig. 5). The branch then increases in length without further increasing in diameter after primary thickening and vertical establishment are completed. This has been termed “maintenance growth” by DeMason^28^. This term is preferred by DeMason and the authors of the present study, as it defines a growth type which lies between the initial primary thickening growth of the branch and not yet occurring secondary thickening growth by means of a STM of the branch. A clear distinction between branch STM and PTM is not possible. Rudall^41^ has reviewed the relationship of PTM and STM and discusses opposing opinions on the longitudinal continuity of both meristems in the taxa *Dracaena*^41,42^. In case of the branch of *D*. *marginata* the meristem expansion of the bud (OS2) establishes a continuity of bud meristem (PTM) and STM of the main stem. This continuity is maintained throughout the development (Figs 5 and 6). However, it cannot be answered satisfactorily whether a branch specific STM is developed below the bud apex during our long-term (180 days) experiment. It can be assumed that a bud STM forms around 90 days after decapitation as branch regions, which are developed by then, do not contribute to the change in orientation. In addition, secondary thickening in the branch insertion region typically accompanies the primary thickening growth of the branch (OS5) to assure vascular connectivity to the increasing number of primary vascular bundles in the branch^31^. For a final answer, however, detailed histological analyses of anatomical sections of these regions are necessary. Comparable experiments on natural branchings of *D*. *marginata*, which are induced after inflorescence or by leaning of the main stem^40,43^ or branch-stem-attachments of monocots lacking a secondary thickening meristem (STM) could additionally contribute to a better understanding of the ontogeny of lateral organs in arborescent monocots.

In summary, MR based analyses of the complex branch ontogeny of *D*. *marginata* provides novel insight in the general ontogeny of lateral organs in arborescent monocots. The understanding of the complexity of branch development has been increased by identifying distinct ontogenetic stages, which is a first approach towards an abstraction and implementation of the branch development into novel bio-inspired fabrication processes and products. This will lead to a transfer of the load-adapted tissue structuring to bio-inspired technical fiber-reinforced ramifications which are of increasing interest in different fields of technology and in building construction.

## Material and Methods

### Plant material and preparation

Individual plants of *Dracaena marginata* originated from commercial nurseries and were kept in the greenhouse of the Botanic Garden of the University of Freiburg.

Five plant specimens that were comparable in size (height: 52–56 cm; stem diameter: ca. 2,5 cm) and habitus (straight erect axial shoot with branching restricted to the apical region) were selected for the experiments using magnetic resonance imaging (MRI). Each plant was decapitated directly underneath the apical branching region of the main stem. This induces the germination of dormant buds leading to novel branches^15,43^. The surface of the cut was immediately sealed using melted candle wax, as it does not affect MR images and prevents infections caused by pathogens or rapid drying effects, both of which can be lethal for the plant. Subsequently, the first ten dormant buds located underneath the decapitation site were marked and numbered consecutively from apical to basal direction using a permanent marker. Finally, one specimen was chosen and taken to the MR imaging facility (Medical Physics, Department of Radiology, University Medical Center Freiburg), where its roots were freed from the soil prior to image acquisition. The size of the field of view during MR imaging allowed for analysis of the most apical four buds (B1–B4).

Apart from preparations for MR imaging experiments, two branch attachments of another specimen with a branch diameter of 1.5–2 cm were dissected and trimmed into size for preparing tangential (axial) and longitudinal (sagittal) sections of the branch-stem-attachments. These sections were used for comparison of magnetic resonance and light microscopy images (Fig. 1a,b) and did not serve for defining ontogenetic stages.

### Magnetic resonance imaging (MRI)

The apical stem segment of the selected plant was imaged immediately after decapitation (day 0) and again after 4 days (day 4). Hereafter, imaging was repeated every five days until day 50. The plant was then imaged at day 65 and again at day 90. Subsequently, the developing branches were imaged every 30 days until day 180. To maintain information on the orientation of the sample, a marker was attached to the apical stem segment. This marker was used as a rough positional reference prior to a precise alignment of the images.

Magnetic resonance imaging was performed on a 9.4 T Bruker Biospec 94/20 small animal scanner. A 3D FLASH sequence (gradient echo) was chosen for high-resolution 3D imaging as it provides a suitable signal-to-noise ratio (SNR) despite the long acquisition time necessary for image acquisition^2,44^. A quadrature volume coil with an inner diameter of 3.8 cm was chosen within the first 35 days before a same type coil with an inner diameter of 7 cm was used to account for branch growth. Accordingly, the field of view (FOV) for the 3D images was adapted and ranged from 5 × 5 × 3.6 cm^3^ (matrix size 500 × 500 × 360) to 8 × 8 × 4 cm^3^ (matrix size 800 × 800 × 400). The number of averages (NA) was between 5–14 to ensure that all 3D data sets had comparable signal-to-noise ratios (SNR) and a total imaging time of approx. 12–14 h. Further imaging parameters were: image resolution: 100 × 100 × 100 μm^3^; repetition time (TR): 26 ms; flip angle: 17°; echo time (TE) was in the range of 6.5–7.1 ms. The parameters for day 90 deviated, as the resolution was 150 μm and the flip angle 13°.

### Image post processing

An inevitable post processing step is the image registration. Since the plant has to be extracted from the MR scanner after every acquired image, the orientation of the sample cannot be maintained exactly identical during every image repetition. The attached marker serves as an approximate reference in early ontogenetic stages when buds are still small and hidden within the stem tissues. With proceeding development, the branches served as approximate references. The registration of 3D images of developing monocot plant structures is challenging. It was of central importance to ensure an accurate alignment of the numerous vascular bundles especially within the developing branch-stem-attachment region. However, complications arise from ontogenetic translations and deformations of the outer and inner arrangement of the plant due to growth of branch and stem. The accuracy of applied automated registrations was insufficient due to a strong self-similarity of the various vascular bundles. Therefore, a careful and time consuming manual registration of each dataset to the reference data was conducted using the transforms module of 3D-Slicer Version 4.3.1^45^. Distinct and retrievable inner tissue features (wounds, bud or leaf traces, stem indentations etc.) at three positions within the imaged stem region were chosen for the fine tuning of the image registration.

3D Slicer was also used for manually segmenting the 3D structure and interconnection of bud specific vascular bundles of the imaged buds at day 0 using the paint effect of the editor module. This was necessary for analyzing the initial connection of the bud to the main stem (bud and leaf traces) and to identify meristematic tissues: PTM, STM and apical meristem. For the 3D reconstruction of distinct ontogenetic stages, the lateral meristem and the newly developed vascular system of the branch were segmented semi-automatically using the active contour segmentation (snake) mode of ITK-SNAP Version 3.0.0^46^. The segmentation images were then exported as NIFTI Files (.nii) and imported into 3D Slicer to create quasi 3D data representations of the tissues according to the procedure described in Hesse *et al*.^2^.

### Image artifacts

A variety of image artifacts occurred during magnetic resonance imaging and needed to be addressed to ensure sufficient image interpretation. The most prominent artifacts are signal voids in the periphery of the FOV (e.g. Supplementary Fig. S8a) and distortions outside of the isocenter of the magnet resulting in effective resolution loss (see bud B4 in Fig. 4 and Supplementary Figs S6 and S7). The signal voids are typical for inhomogeneities of the B1 excitation field or for short T2*-values resulting from the magnetic susceptibility differences within the plant material, caused e.g. by air spaces in the plant tissues. Image distortions can be explained by non-linearities of the imaging gradients. The partial volume artifact (partial volume effect or partial volume problem^21,47^) particularly affected regions in which different tissues are densely packed or dedifferentiation occurred resulting in a blurry appearance within these regions (see Supplementary Fig. S8b,c).

### Meristem terminology

The primary thickening meristem (PTM) is separated from the nearby apical meristem. PTM is located within the periphery of a stem or branch and extends in a downward direction^28,39,41,48,49^. Zimmermann and Tomlinson have used the terminology “meristematic cap” to describe the meristematic tissues in close proximity to the apical meristem and delimit it from the PTM^22,27,28,34,41,43^. In the present study, the term “meristematic cap” was used to describe the most apical section of the PTM and therefore was considered being a distinct region of the PTM. As it is not possible to distinguish PTM and apical meristem in young developing buds in MR images this region is termed bud meristem. The secondary thickening meristem (STM) increases the thickness of stems and branches^31,39,41^. PTM and STM were continuous between branch and main stem in *D*. *marginata*. A transition of both meristems was not visible, which is why the terminology lateral meristem was used in cases when their distinction was not possible. In accordance with Jura-Morawiec *et al*.^39^ the term ‘storied cork’ referred to the monocot meristem responsible for the development of secondary protective tissues (cork^39,50^).

### Branch-stem-angle

The angle between branch and stem was determined on the basis of sagittal MR images at the adaxial side of the branch. For this, two tangents were placed along specific features in the MR images: one tangent was placed along the PTM of the branch in close proximity to the crown. The second tangent was placed along the STM of the main stem. The branch-stem-angle was then determined at the intersection of both tangents. The angles could only be determined for the buds B2 and B3 as these were the only buds that fully developed.

### Light microscopy (LM)

Larger samples of branch-stem-attachments (branch diameter <1 cm) of *D*. *marginata* were macerated for two weeks in an ethylenediamine solution (10%^51,52^). The samples were then infiltrated with polyethylene glycol 2000 (PEG 2000) for one month to ensure that all tissues were embedded. The unstained sections were imaged using a light microscope (Olympus BX61, Olympus Europa GmbH, Hamburg, Germany) and the software Cell^P^ 2.8 (Olympus Soft Imaging Solutions GmbH, Münster, Gemany).

### Data availability statement format guidelines

The datasets generated during and/or analysed during the current study are available from the corresponding author on reasonable request.

## Electronic supplementary material

Supplementary Information
