# Supplementary material for: A qualitative analysis of the bud ontogeny of Dracaena marginata using high-resolution magnetic resonance imaging
Source: Sci Rep. 2018 Jun 29;8:9881. doi: 10.1038/s41598-018-27823-1 (PMC6026186; doi:10.1038/s41598-018-27823-1)
Supplement: Supplementary file 1 — Supplementary Information [file 41598_2018_27823_MOESM1_ESM.pdf]

Supplementary Information

A qualitative analysis of the bud ontogeny of *Dracaena marginata* using high-resolution magnetic resonance imaging

Linnea Hesse[1,2,*], Jochen Leupold[3], Thomas Speck[1,2], Tom Masselter[1,2]

[1]Plant Biomechanics Group and Botanic Garden, University of Freiburg, Germany.

[2]Freiburg Center for Interactive Materials and Bioinspired Technologies (FIT), Germany.

[3]Department of Radiology, Medical Physics, Medical Center University of Freiburg; Faculty of Medicine, University of Freiburg, Freiburg, Germany.

*Corresponding author: linnea.hesse@biologie.uni-freiburg.de

Supplementary Figures

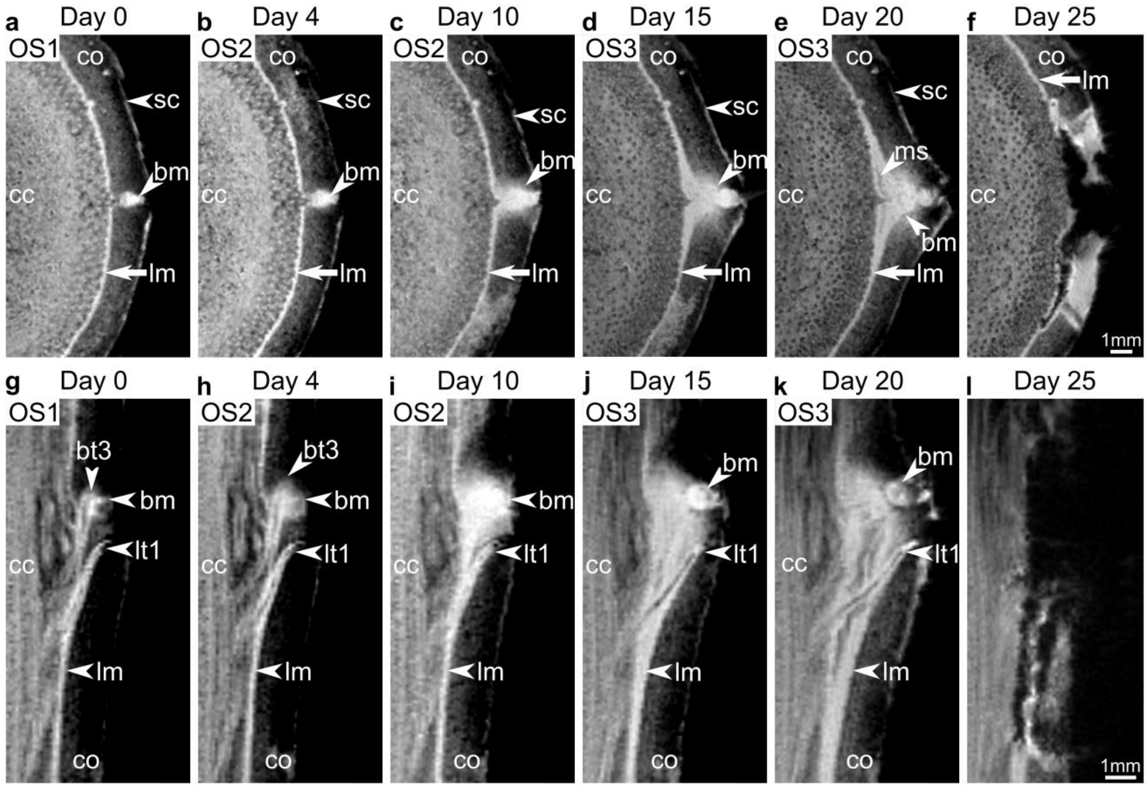

Figure S1: Magnetic resonance images of the ontogeny of bud B1 from day of decapitation until its dying back 25 days after decapitation (day 25). The developmental stages (OS1-OS3) are given for each image panel. a-f) axial images of B1. g-l) sagittal images of B1. The bud is initially embedded within the cortex (co) of the main stem and the bud meristem (bm) is supplied by three bud traces. Only bt3 is depicted as the other bud traces are not included in the image plane. The leaf trace closest to the bud (lt1) is given in the sagittal images, lt2 is not included in the image plane. bm: bud meristem; bt3: bud trace nr. 3 (also see Figure 5); cc: central cylinder of the main stem; co: cortex; lm: lateral meristem; lt1: leaf trace nr. 1 (also see Figure 5); ms: meristem splitting; sc: storied cork.

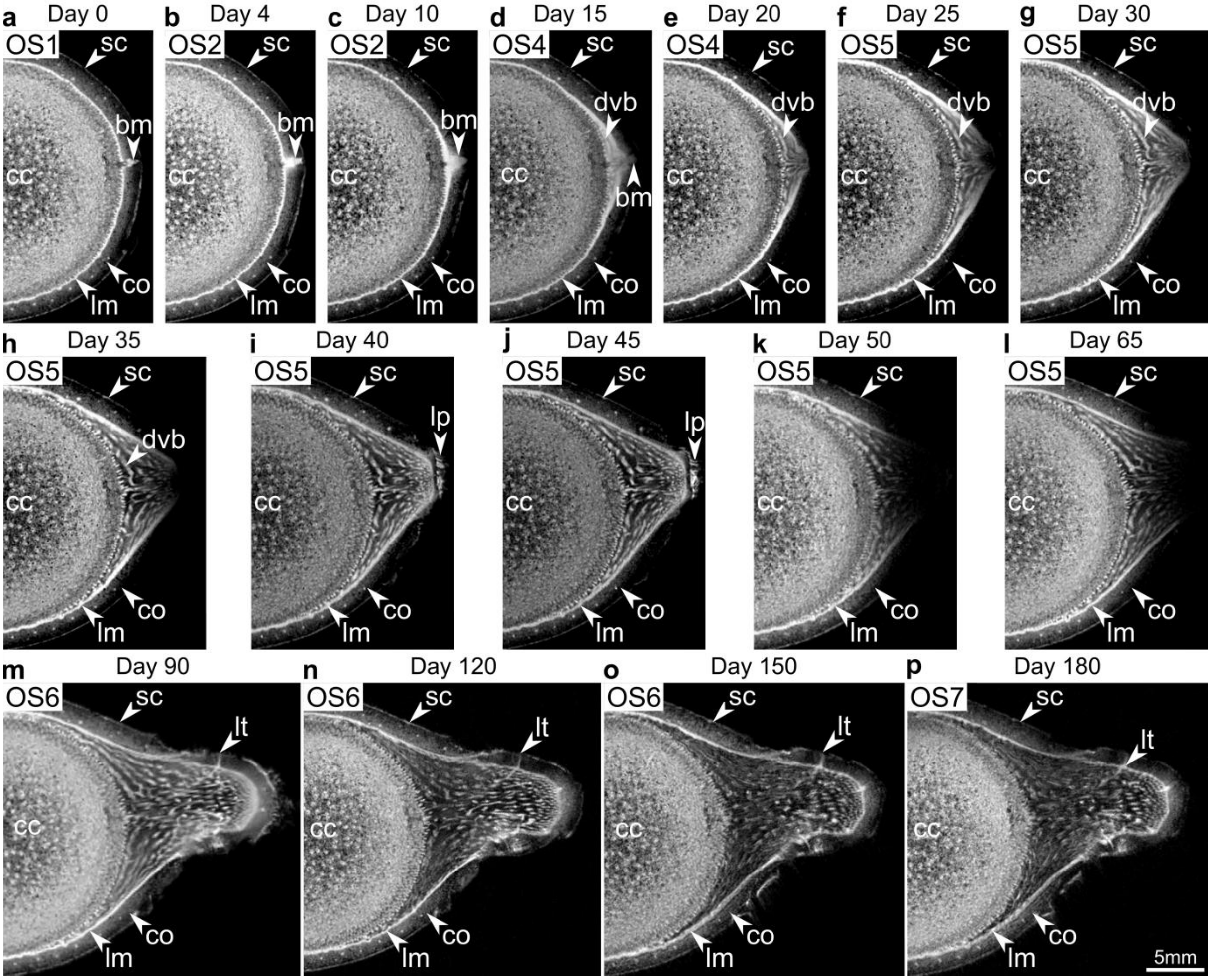

Figure S2: Magnetic resonance images (axial) of the ontogeny of bud B2 from day of decapitation until 180 days after decapitation (day 180). The developmental stages (OS1-OS3) are given for each image panel. The bud is initially embedded within the cortex (co) of the main stem. Image artifacts appear as dark shadows concealing the apex of the branch in images of day 20-25, days 30, 35, 50 and 65. bm: bud meristem; cc: central cylinder of the main stem; co: cortex; dvb: developing vascular bundles; lm: lateral meristem; lp: leaf primordium; sc: storied cork.

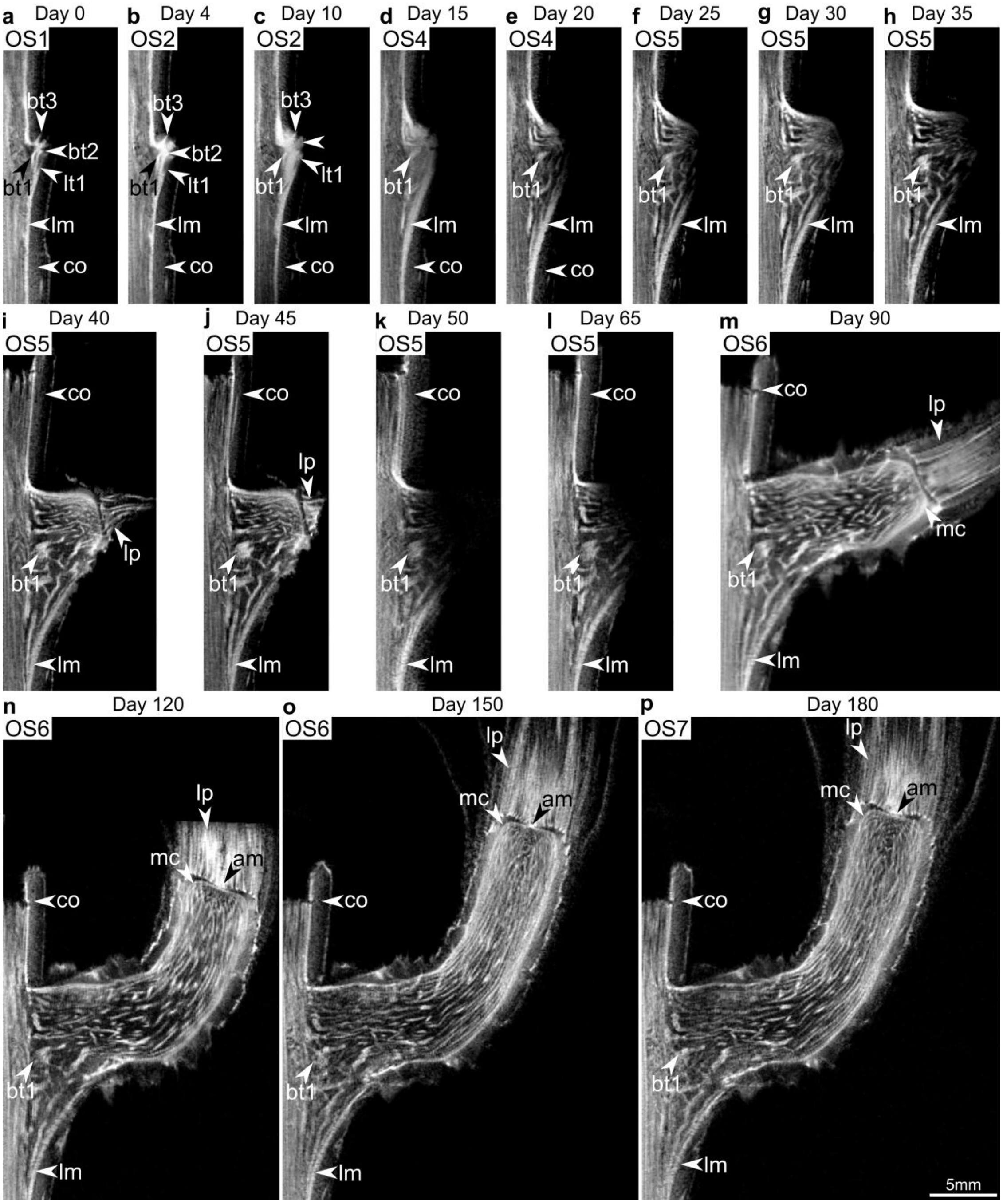

Figure S3: Magnetic resonance images (sagittal) of the ontogeny of bud B2 from day of decapitation until 180 days after decapitation (day 180). The developmental stages (OS1-OS3) are given for each image panel. The bud is initially embedded within the cortex (co) of the main stem. The bud traces (bt1-bt3) and the leaf trace lt1 are visible. Image artifacts appear as dark shadows concealing the apex of the branch in images of day20-25, days 30, 35, 50 and 65. am: apical meristem; bm: bud meristem; bt1-3: bud traces; lt1: leaf trace; co: cortex; lm: lateral meristem; lp: leaf primordial; mc: meristematic cap.

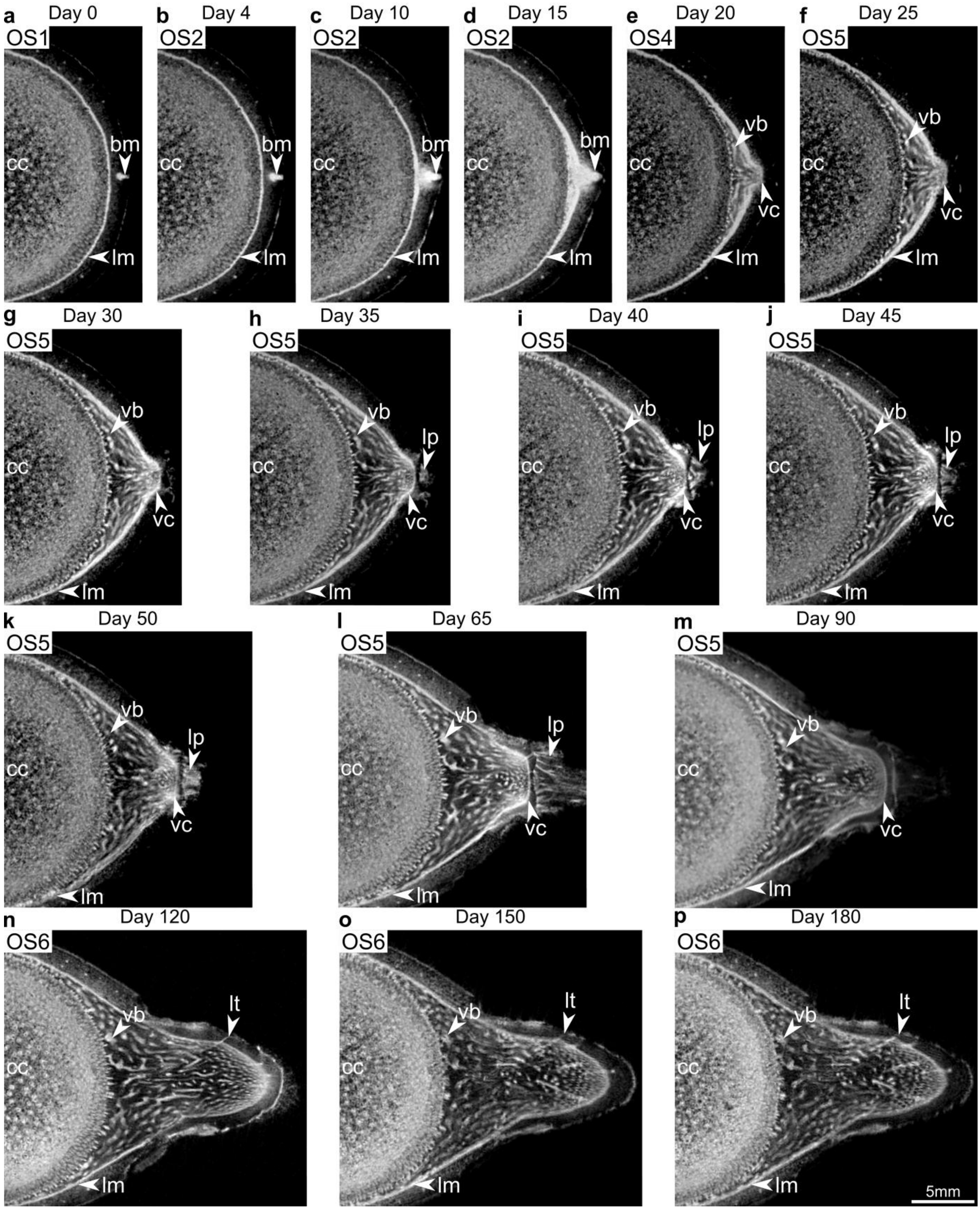

Figure S4: Magnetic resonance images (axial) of the ontogeny of bud B3 from day of decapitation until 180 days after decapitation (day 180). The developmental stages (OS1-OS3) are given for each image panel. Leaf primordia (lp) are successively increased in size. bm: bud meristem; cc: central cylinder of the main stem; lm: lateral meristem; lp: leaf primordia; vb: vascular bundle with fiber cap; vc: apical vegetative cone of the branch.

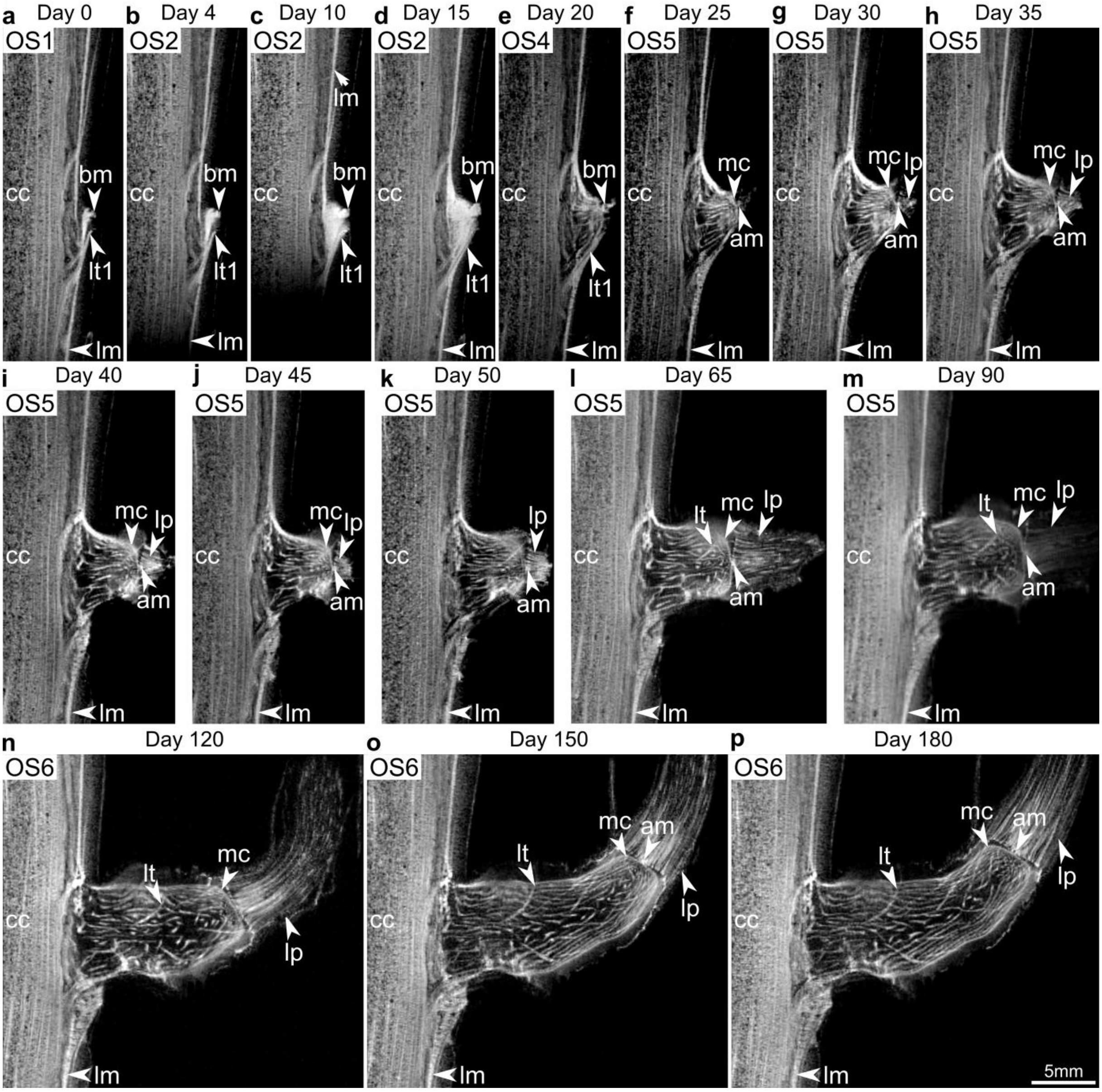

Figure S5: Magnetic resonance images (sagittal) of the ontogeny of bud B3 from day of decapitation until 180 days after decapitation (day 180). The developmental stages (OS1-OS3) are given for each image panel. am: apical meristem; bm: bud meristem; cc: central cylinder of the main stem; lm: lateral meristem; lp: leaf primordia; lt: leaf trace; lt1: specific leaf trace in closest proximity of the bud; mc: meristematic cap; vb: vascular bundle with fiber cap; vc: apical vegetative cone of the branch.

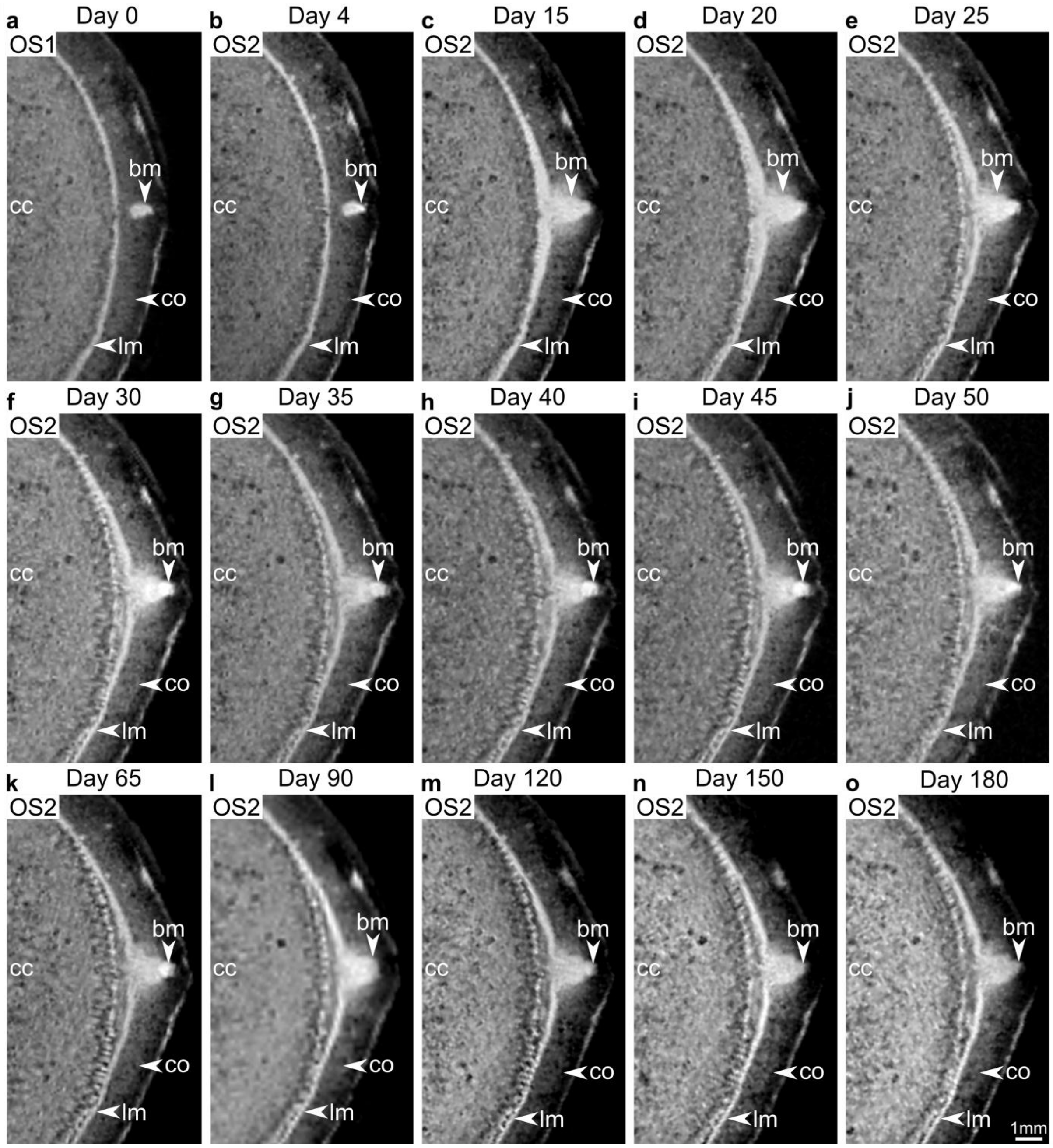

Figure S6: Magnetic resonance images (axial) of the ontogeny of bud B4 from day of decapitation until 180 days after decapitation (day 180). The developmental stages (OS1-OS2) are given for each image panel. bm: bud meristem; cc: central cylinder of the main stem; co: cortex; lm: lateral meristem.

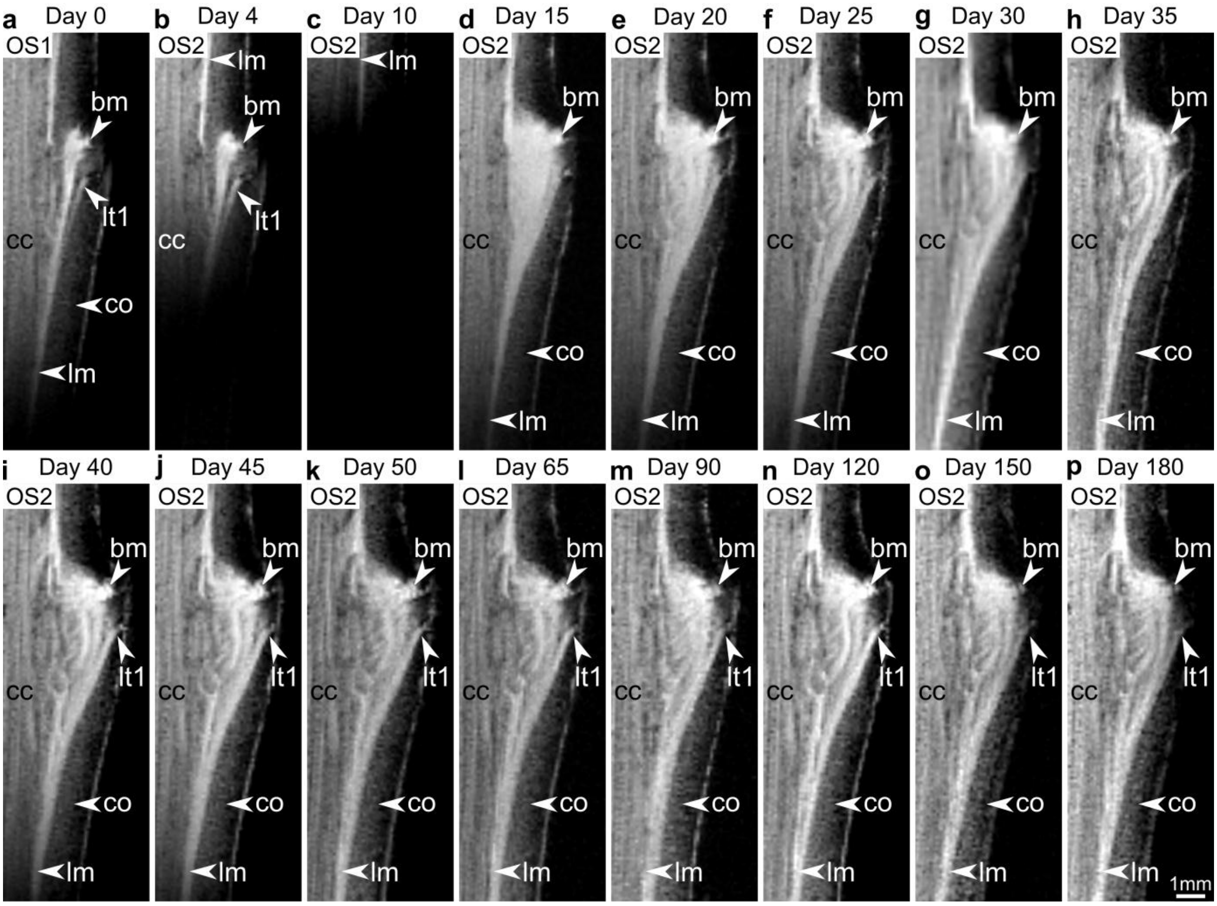

Figure S7: Magnetic resonance images (sagittal) of the ontogeny of bud B4 from day of decapitation until 180 days after decapitation (day 180). The developmental stages (OS1-OS2) are given for each image panel. The gradient non-linearities led to image distortions outside of the isocenter of the magnet resulting in effective resolution loss at day 10. bm: bud meristem; cc: central cylinder of the main stem; co: cortex; lm: lateral meristem.

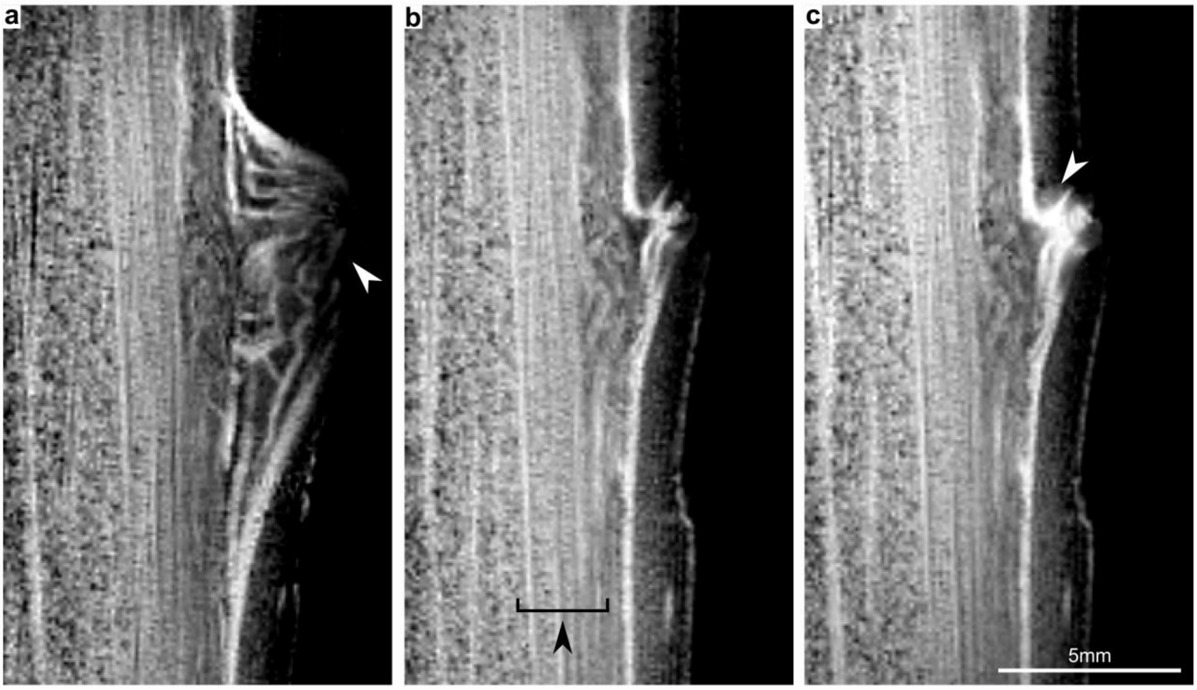

Figure S8: Typical image artifacts that occurred during the experiment. a) Signal voids located in the periphery of the sample (i.e. the periphery of the FOV). b) The black arrow and bar highlight the blurry appearance of peripheral stem regions due to increased tissue density. c) partial volume- and possibly motion artifacts affecting young proliferating tissue regions (here: the young bud, white arrow).
